# Sociodrama as a “potential stage” for creating participative and transformative research on social work with families living in vulnerable situations

**DOI:** 10.1007/s11620-020-00563-z

**Published:** 2020-10-20

**Authors:** Marco Ius

**Affiliations:** grid.5608.b 0000 0004 1757 3470 Dep. FISPPA, University of Padua, Via Beato Pellegrino, 28, 35137 Padova, Italy

**Keywords:** Psychodrama, Sociodrama, Psychodrama research, Participation, Social work, Work with Families, Psychodrama, Soziodrama, Psychodramaforschung, Partizipation, Sozialarbeit, Arbeit mit Familien

## Abstract

This article of the Zeitschrift für Psychodrama und Soziometrie aims to explore how Morenian sociodrama and its techniques represent valuable tools for participative research with social professionals and teachers working with children and their families in vulnerable situations. After introducing the Canon of Creativity by Moreno and its connection to art-based research, sociodrama is theoretically introduced and proposed as a research tool. This is followed by a description of an experience of participative research with a group of roughly 40 professionals within the national Italian programme P.I.P.P.I. The process of the session is described and discussed, in order to demonstrate how sociodrama can be an interesting tool for social research. The conclusion provides connections between practice and research, proposing the involvement of families in future activities, and highlighting possible future theoretical explorations in order to examine the topic in greater depth.

## Abstract (de)

In diesem Artikel der Zeitschrift für Psychodrama und Soziometrie soll untersucht werden, wie das Soziodrama nach Moreno und seine Techniken wertvolle Werkzeuge für die partizipative Forschung mit SozialarbeiterInnen und LehrerInnen darstellen, die mit Kindern in vulnerablen Familien arbeiten. Nach der Einführung der Theorie der Kreativität nach Moreno und seiner Verbindung mit kunstbasierter Forschung wird das Soziodrama theoretisch als Forschungsinstrument vorgestellt. Darauf folgt eine Beschreibung der Erfahrung partizipativer Forschung mit einer Gruppe von ungefähr 40 Fachleuten innerhalb des nationalen italienischen Programms P.I.P.P.I. Der Prozess der Sitzung wird wiedergegeben und diskutiert, um zu zeigen, wie Soziodrama als ein interessantes Instrument für die Sozialforschung verwendet werden kann. Die Schlussfolgerung stellt Verbindungen zwischen Praxis und Forschung her, schlägt die Einbeziehung von Familien in zukünftige Aktivitäten vor und regt mögliche zukünftige theoretische Untersuchungen an, um das Thema eingehender zu untersuchen.

## Moreno, creativity and research: an introduction

The legendary biologist Edward O. Wilson launching his book on “The origins of creativity” wrote “Creativity is the unique and defining trait of our species; and its ultimate goal, self-understanding” (Wilson 2017). With this opening, he begins the examination of humanities and their relationship to sciences.

The search for understanding about human aspects and finding possibilities and modalities for the expression and fulfilment of each person, is conceived and proposed by J.L Moreno not just as a mere cognitive process, but as a process connecting cognition and the practice of action within the social context each person lives in. Creativity has a crucial role in this process. It is, together with spontaneity, considered the cornerstone of Moreno’s theory that underpins the development of sociometry, psychodrama and sociodrama (Moreno 1953).

In “*Who shall survive?”* Moreno affirms *“*creativity belongs to the categories of substance—it is the arch substance—spontaneity to the category of catalyser—it is the arch catalyser” (1953, p. 40). As a physician and social philosopher, he picked out these categories from his studies on metaphysics and, through his method, he transferred them from the philosophical level to an empirical one. In doing so, he began to research and elaborate a methodological proposal of connecting theory to practice in which “action” is the key element to understanding and for the development of each human being, communities, and the world, to fight social injustice and inequality, and to promote the full expression of the potentials and talents of each person (Nolte 2014). The result of a meeting between spontaneity and creativity is called *conserve*, which can be social, technological, or cultural. Conserve is meant in a dual modality: it represents the result of the encounter between spontaneity and creativity and the base on which to start a new creative process. Consequently, all productions, since they are results of a human creative process, represents a conserve (inventions, tools, social institutions, codified practices, stereotypes, works of art, etc.), and when they are subsumed within the approach of innovation are a starter to a further production. However, when a conserve is deified and rigidly taken as unchangeable, the creativity process is interrupted, and development is undermined. A similar process was conceptualized as the stage of “generativity—stagnation” by Erik Erickson in his “cycle of life” development theory (Erikson and Erikson 1998) that, although particularly referred to adulthood, seems to be related with the “Canon of Creativity” by Moreno (1955).

### Moreno and “action research”

Moreno’s approach, when considered from a research perspective, is meant as an action theory that is the result of a creative integration of theories, research, and practice. This approach considers the researcher “as ‘co-operator’ just as the researched are ‘co-actors’” (Gunz 1996, p. 146). The common goal of researcher and co-actors is to become “experts” of human connection in order to enable communities to shift from a non-caring and individualistic attitude to the attitude of connection, care, love, and promotion of the development of all the members and the community as a whole (Moreno 1953). For this reason, referring to Moreno just as the father of psychodrama seems to be limited. His approach can be considered not only as strictly therapeutic, from the psychological and psychiatric side, but also, and even more so, as a social, pedagogical, and educational method that, by offering each person the possibility to unfold his/her spontaneity and creativity, builds communities of equity and support.

Therefore, the approach of Moreno is useful to other more commonly used frameworks on “action research” based, *in primis,* on the work by Kurt Lewin (1946) and on the influences the pragmatic and experiential approach in education by John Dewey (1938). Gunz acknowledges Moreno as a founder, or at least a pioneer, of action research (1996) underlying how Lewin’s achievements are rooted in sociometry.

What is key, in Moreno, is that action research deals with the integration and participation of people in the process of social change and in the fight against social inequalities.

Chaskin defines “community capacity” as the interaction of different capital (human, organizational and social) within a given community and he highlight that that capital can be used and leverage to solve problems and to promote the well-being of the community itself (Chaskin 2001; Chaskin et al. 2001). Looking at community capacity from a Morenian perspective and within the field of action research, connects Morenian thought to the perspective of critical pedagogy by Paulo Freire (1970) who claims education is the key to liberate people from oppression by establishing an egalitarian power shift in education and social processes. From a sociometry perspective, what happens on the stage via sociodrama and psychodrama fosters what Freire considers the achievement of critical consciousness of social structures. This empowers participants with the ability to explore how they can contribute, from their different roles, to social change.

Action research is defined as a research “grounded in lived experience, developed in partnership, addressing significant problems, working with, rather than simply studying, people, developing new ways of seeing/theorizing the world, and leaving infrastructure in its wake” (Bradbury and Reason 2003, p. 155). Hence, social workers operate as “frontline implementers of important social policies and suggest how action research can be used to both implement and also influence the creation of such policies” (p. 155).

### Creativity, art-based research, and sociodrama

Including the notions of spontaneity-creativity-conserve, within a research perspective, attention is given to the role of expressive arts in research and to Art Based Research (ABR) which from a methodological point of view refers to “qualitative research paradigms, such as narrative, hermeneutic, heuristic, and phenomenologically based perspectives, as art making in all its modalities was conceptualized as a form of direct experience” (Childton and Leavy 2014, p. 405). ABR represents an opportunity for “knowing different and collaboratively” (Liamputtong and Rumbold 2008) in a way that is intrinsically “socially responsible, politically activist and locally useful” (Finley 2005, p. 681). Using artistic expression may appear to be “frivolous, trivializing, or eccentric. There may be an assumption that the aim of arts work is emotional expression at the expense of analytic understanding” (Walton 2012, p. 724–725). However, it is strongly connected with social work practice because it implies the ability to communicate and to find different and unusual ways of communication and connections to be efficient, meaningful and effective. ABR fosters processes aimed at avoiding to suppress the subject to a passive role by promoting participation of each actor and by listening to the different voices and perspectives according to an ethical frame that connects relational practices to the sought after social justice. Doing ABR implies using methods and tools belonging to creative arts field “in order to address the social research questions in holistic and engaged ways in which theory and practice are intertwined” (Leavy 2015, p. 4). These tools are intentionally chosen and used in one or more phase of the research process: data generation and collection, analysis, interpretation, and presentation and discussion.

I suggest, in this specific reflection on research, to consider the Morenian use of “drama” and theatre as a form of expressive art (Moreno 1947) and, particularly, to refer to sociodrama as relevant method to use Morenian “drama” in a research perspective. Moreno influenced and inspired by the “philosopher of the encounter” Martin Buber, focused on the meeting between two people as the smallest unit of relationship that is the tenet to each human development. From this unit, he developed his contribution to role theory and group theory, with many consequent implications for the practice: the goal of each practice is to promote each person being, at the same time, the creator of his/her development and the co-creator of the community, which implies fostering the development of other members. Sociodrama is an educational modality that, by creating “for instance situations”, “directs its attention to human growth and interaction by attending to collective role aspects. It also promotes human development in a global manner […] actively immerses the participants in the process [… *who is*] involved, activated, and impacted by the group process as it creates enactments of importance to all” (Sternberg and Garcia 2000, p. 7). Role is referred to as a set of behaviours culturally recognized and agreed upon, and it is considered in its two dimensions: one shared and defined by collective components, and the other individual and defined by private components (Moreno 1947, 1961). Contrary to psychodrama, which facilitates the exploration of private components focusing on intrapsychic aspects (clinical—therapeutic goals), sociodrama is a group action method that focuses on group and social components (Moreno 1953), and offers participants a safe method and context to work on the roles they share with others by sorting out ideas, making decisions, empowering the way they play roles, practicing new roles, and becoming more spontaneous and playful.

## Objectives

Since social research may be considered as a form of searching for understanding about human aspects, this paper will explore and discuss if and how creativity can contribute to the research process by assuming a Morenian perspective (spontaneity, creativity, conserve). More, it aims at exploring if and how sociodrama and respective techniques are useful and appropriate tools for a participatory process connecting research, training, and intervention in groups of social professionals working with vulnerable families. Starting with these aspects, the goal of the paper is to present and discuss the rationale, structure, and process of a participatory and transformative research meeting with a group of roughly 40 social professionals and teachers involved in the Territorial Laboratory of the programme P.I.P.P.I. in a local authority of a city of Southern Italy.

### P.I.P.P.I. and the LabT

P.I.P.P.I. is a research-training-intervention programme that was developed by the Italian Ministry of Labour and Social Affairs, in cooperation with the Laboratory of Research and Intervention in Family Education (LabRIEF), at the University of Padua (Italy) (Fantozzi et al. 2014; Ius 2020a; Milani et al. 2016; Santello et al. 2017, 2018; Serbati et al. 2016; Zanon et al. 2016; consult these papers for a wider presentation of the programme). Since 2011, P.I.P.P.I. has been run by Regional and Local welfare services across Italy as an intensive programme to support families living in vulnerable situations and facing child neglect (Lacharité et al. 2006; Sellenet 2007), and to promote children’s wellbeing through a multi-professional, ecological, and resilience-based intervention (Ius 2020a). The programme has on the whole involved roughly 8000 professionals from Social and Health Services and Schools, as well as 4000 children and their families in more than 200 territories of the 20 regions across Italy.

From 2017, the districts that have implemented the programme for at least 2 years have been given the choice of entering the Advanced Level (AL) of the programme. This level requires them to focus and organize their implementation task on two levels. The first regards the work according to the Base Level implementing the programme with a minimum number of 20 families. The second focuses on the organization of the services, by establishing the Territorial Laboratory (LabT) (Di Masi et al. 2019).

LabT is a reflective processing space bringing together stakeholders and professionals of a district working with children and families. According to the Participative and Transformative Evaluation framework underpinning P.I.P.P.I. (Serbati 2017; Serbati and Milani 2013), the LabT has 3 main goals:

- To reflect on and use qualitative and quantitative research data from previous implementation activities that the scientific group of the university provides through a dossier each LabT receives.
- To organise meetings with professionals, families, and other actors where they self-evaluate service practice, discuss and reflect on, starting from the dossier in order to define a “research question” or a relevant topic to deepen and to plan a future step of innovation within and for the district.
- To plan and run training sessions for professionals involved in the work with children and families, with the specific involvement and help of three professionals who attend a specific training for local trainers organized by the university.

Regardless of the professional role of each participant to the LabT, each of them participates in a “stakeholder” role because everybody is involved in the process aimed at developing the knowledge by participative research that results in action for innovation, social change and social promotion (Chevalier et al. 2013).

## The research meeting with professionals: context, subjects, and goals

This paper will examine the local authority A1 of Ariano Irpino, Campania Region, Southern Italy, consist of a group of 29 municipalities spread in a hilly and mountain territory of 821 sq km. It counts about 90,000 inhabitants, most of them living in the lead municipality Ariano Irpino (about 22,000 inhabitants) and other two municipalities (about 8000 each), and the rest of the population lives in small municipalities (less than 4000), including a few cases of very small centres (less than 800).

Social welfare is led, organized and managed by a Consortium that gathers all professionals working with people in the services spread withing the territory.

The local authority took part in 4 editions of the implementation of the programme P.I.P.P.I. (2013–14, 2017–18, 2018–20, 2019–21) cumulatively including in it 71 families and their 73 children.

Since 2017, the programme has been implemented at the advanced level. Besides the work with each family, the advanced level has been planned and implemented to empower the governance practices within the territory with particular focus on fostering processes that allow social services and schools to make decisions and organize their actions so that community relations developed within and between the ecological systems of children, families, and communities (Bronfenbrenner 1979, 2005).

For this reason, the P.I.P.P.I. leading group promoted, step by step, an intense participation of teachers, including both the ones working directly with children belonging to families living in vulnerable situation and others.

Data about the children and families^1^ show families face vulnerability regarding economical condition-work (70% of families), poor education of parents (50%), psychological difficulty of parents (45%), disability or special needs of children (41%), housing (38%), social isolation (30%), and traumatic event (30%). The programme was mostly addressed to children of 6–10 years of age (65%) and less used with children under 5 (13%) and more than 11 (22%).

Almost all the children are Italians (90%) while only 10% are not Italian citizens, since they were born abroad from no Italian parents. The children live in household with both parents (55%), with a single parent (32%), or in kinship care or residential care (13%) when the programme was used to foster the process of family reunification.

As previously mentioned, the advanced level was entered with the intent of improving cooperation between social services and schools at a communitywide level and to involve teachers working with children involved in the programme.

The first two-year actions (2017–2018) involved social service providers and professionals and aimed at involving teachers in the implementation of P.I.P.P.I. with children and their families, engaged with social care due to their needs. The local meeting with researchers at the university aimed at collecting teachers’ perspectives and inviting them to take part in the training run by LabT members. The training was intentionally located in schools as an effort to encourage the relocation of social services towards schools. It was attended by 50 teachers belonging to the 14 different public schools working with children 3–14 years old living in the 29 municipalities. As a result of the training, three teachers, in agreement with their principal, wanted to be more involved and become promoters in their institutions concerning school-social services cooperation.

In the second iteration of the advanced level, the participation of those teachers in the training of trainers provided by the University, made the previous group of trainers stronger and more multidisciplinary. As a result, the activity for the LabT planned to gather teachers and professionals to reflect and research together on the strengths, challenges, and development areas of their mutual work with children and families.

Starting with the concerns identified in the dossier, the LabT organized a daily session to collaboratively research the following questions: What worked/did not work in the care pathway with families? What were the key factors in the school-services collaboration? How could social services be more helpful and supportive towards the school and vice versa? How do families participate in the processes? Is it meaningful and possible for social services to organize school interventions targeted at all children to promote wellbeing for all and not only for those in the care system? What are the understanding and culture of school, services, and the community about this? What is the role of the families in this? All the families, the one in care and the others?

## The research technique: from focus group to sociodrama

In the process of planning the day’s structure, the LabT proposed organising a focus group to explore the questions above. The high level of interest and participation of teachers and professionals resulted in a group of about 40 people. The focus group process would have allowed a meeting with a minimum number of participants, generating into a conflicted situation of choice and exclusion, which is not useful to the participatory attitude of P.I.P.P.I. Since this aspect would not have fostered community building between participants, other techniques appeared to be necessary. Sociodrama was selected:

- Owing to the presence of different professionals, expertise, and multiple roles: social workers, professional home carers (educators trained in social pedagogy), teachers, school headmasters, the director of the social service, some of whom are both colleagues and peer-trainers.
- For the goals of the LabT that consist in a co-productive contest where, as previously presented, participants (professionals and university researchers) are requested to assume the symmetrical relation of being *co-researchers*, rather than the asymmetrical relation between *researcher* and *researched*.
- Since, for many professionals the meeting represented a continuation of the training and the work they had been involved in within the programme, sociodrama is a crucial tool to promote team building and networking, fostering the belonging to the community of professionals, by exploring the topics in an active way.
- To allow participants to look at the issue from several roles and thus to explore it through the eyes, feelings, and thoughts of many. Sociodrama fosters a process enabling participants to experience the whole system and becoming aware of it.
- To unlock the creativity needed to generate new choices and new ideas for the continuation of the LabT.

### Sociodrama techniques

The techniques used during the sociodrama were the following:

- Soliloquy: “form of discourse in which a character talks to himself or reveals his thoughts in order to form a monologue without addressing a listener” (Sternberg and Garcia 2000, p. 65) and to let the actor externalize the hidden feelings and thoughts by “thinking out loud” (Cruz et al. 2018)
- Multiple doubles: the double is when “the auxiliary ego plays the role, or an aspect of protagonists’ role, by standing to the side or behind him/her; expressing the protagonist’s unspoken thoughts and feelings” (Cruz et al. 2018, p. 6). Multiple doubling means more than one participant to double for an actor (Sternberg and Garcia 2000, p. 64). It is particularly useful in sociodrama to ask for a volunteer doubling to expand the group social knowledge, feelings, and actions on a particular role, and its relevant topic.
- Sculpture of a future projection: a combination of sculpture that is “a concretization in which enactors create a living tableau of interrelationships” with a future projection that consists in setting the scene in the future to see how things are and to perform the desirable situation to work out lately how to fill the gap between the two times or which actions need to be planned to reach the expected outcome (Sternberg and Garcia 2000, p. 69).

### The role of the researcher as a sociodrama facilitator

Researchers have the “ethical imperative to ensure that the ways in which we engage in research with communities honour their wisdom and expertise” and therefore are “obligated to represent research methods and to find ways of ensuring that the implementation of research methods remains rigorous, while also establishing engaging and accessible processes amenable to the communities” they work with (Liebenberg 2018, p. 1–2). Since the interaction of research and action is specifically intended to result in social change, the position of the researcher (Grbich 2004) becomes similar to, and associated with, the director of psychodrama, and even more of sociodrama. However, I prefer to use the term facilitator to underline the disempowerment of researcher and to emphasize his/her heuristic and hermeneutic attitude. The researcher-facilitator does not have the function of guiding the group by holding a higher knowledge but, on the contrary, he/she has the task of fostering a creative group process in which all participants are co-actors and co-creators, starting from the contribution each of them can bring from his/her role repertoire.

The role of the researcher is to facilitate a context where the involvement and sharing-creation of ideas, actions, thoughts, and emotions of each actor is promoted, facilitated, collected, and put together. Through an intersubjective process, the group participants: 1) can recognize and find themselves, and create the connection leading everyone to a more in-depth level of comprehension of a question related to their profession; 2) can enhance the social cohesion within the group strengthen and nourish the interaction-integration dynamic among the members of the group; 3) can analyse, understand, and discuss what the group creates (the collected data) constantly being encompassed in the research, reflection, and development innovation.

## The meeting: structure and the process as data collection

### The structure of the LabT meeting

The meeting was created by adopting the following structure:

- Using sociometric techniques, the participants met and warmed up between themselves and the two researchers-facilitators. This aimed at welcoming each participant, discouraging judgement and criticism, valuing the contribution of everybody, and to warm up in order to let everybody relax and feel comfortable and safe in the context he/she is involved in and to give motivation for the next step of work.
- Different experience and expertise on P.I.P.P.I. training and implementation arose and using that information the group was divided into homogeneous small groups of 4 people.
- Participants were asked to draw their own work timeline highlighting the significant events portraying their work journey with families in order to reflect on the family-school-service relationship. They shared and discussed in small groups the main contents of their timeline and negotiated a group synthesis, eventually presented in plenary.
- First part of sociodrama activity.
- Sharing to take stock and moving forward.
- Second part of sociodrama activity.
- Sharing and conclusions.

One researcher facilitated all the activities and the other took notes transcribing what participants said and documenting the process by taking pictures.

### Results: the process of sociodrama

The researcher facilitator proposed to the group a sociodramatic activity aimed at expressing and collecting the points of view of the different actors, who are involved in working with families in care. Using two empty chairs the facilitator made the group focus generically on:

- The *world* of the social services working with a family living in vulnerable situation, and therefore being committed to collaborating with the school.
- The *world* of the school whose work is addressed to all children and so has a key role for children in need and collaborates with social services.

Referring to the wide *worlds* enabled participants to choose for themselves which voice to give to the different roles that were present.

Research shows that the participation of families is a key factor for good outcomes of care pathways (Berry 2010). Therefore, professionals are required to work out their own power and control dynamics and enable families to take part in the decision-making process in order to promote and empower their full participation and foster their wellbeing (Arnstein 1969; O’Sullivan 2010). Consequently, at the beginning of the sociodrama, the family was intentionally not given a chair leaving professionals with the possibility and responsibility to include family members according to their framework. This aimed at gathering an understanding how professionals make space for the family “on the stage” of school-service collaboration. To frame the situation, the facilitator asked the group to name the child to make the situation more concrete. A 12-year-old boy named John was chosen.

The facilitator invited participants to voice any possible thoughts, ideas, feelings, emotions, and concerns of one the two worlds. The first one who asked to speak was invited to enter the role of school or service sitting on the assigned chair and to express him/herself by a soliloquy. This technique was chosen to let each actor express his/her point of view and the thoughts and feelings that might be held back, and so not to take part to usual relational dynamic risking in getting stuck and freezing the social role and attitude instead of exploring them, while listening to the others’ point of views.

Each actor was invited to express his/her inner world (thoughts and feelings) and the people in the audience to enter on the stage for multiple doubling actors in order to widen perspectives and open new focuses.

Hereafter, the transcription of the sociodrama contents is presented to show the topics and elements of intervention with families that professionals expressed and explored. In case of multiple doubles of the same actor, sentences are separated by a slash.

- School—Teacher: John spends most of the time at school, I have the right to say something about this child and the problems he reveals.
- Services—Social worker**: **the school must do its job and shouldn’t call me all the time complaining about this child and delegating the problems.
- School—Teacher: we are facing many difficulties with John; we need to take joint action/We need a strong service that bridges us, and John’s family/John entered the 6th grade without a special needs certificate.
  (A participant from the audience asks about the role of the family and is invited to take another chair. She takes for granted that she is John’s mother)
- Family—Mother: these people talk about me without asking me! Nobody has ever told me about the certificate. I feel judged.
- School—Teacher: John has a family and therefore the first thing to do is to meet the family and then close the circle with the services.
- Family: as a family I do not see the problem, so what are you calling me for? I do not have any problems/I know about my son’s issues, but I don’t want him to be labelled and that my son be considered different.
- Service—Social worker: I’ve come to school to grasp when we can meet, share, and think about it together.
- School—Teacher: The family needs to be given time to digest the problem.
- Service—Social worker: I could go for a home visit to meet the mother, have a coffee and in the meantime, I see what she is saying to me.
- School—Teacher: I am tired! Tomorrow I will go to headmaster who must understand that the services are not collaborating, neither is the family.
- School—Principal: Wow, how hard it is! On the top of all I have to do, with all the schools, the children, and the teachers. I cannot be overloaded! I cannot take care of everything.
- Family—Mother: I am John’s mom, I accepted the special needs certificate, but they made me waste so much time, sending me here and there, it is exhausting and confusing. Why don’t you do everything at school?

Observing that John had not yet been brought on the stage by the group, the facilitator suggested participants to consider his point of view. Hence, he was introduced by an actor taking a seat opposite the other actors.

**Fig. 1:**
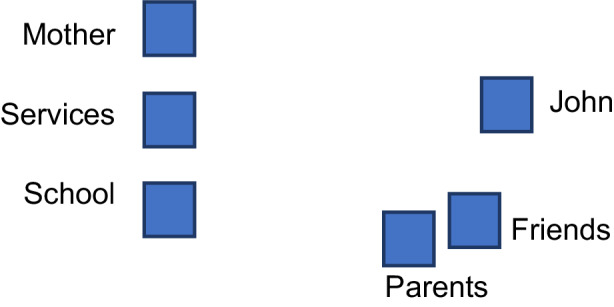
Position of the actors (first part)

- Facilitator: What does John think about what these people are saying? Do you have any clue about it?
- John—the child: they tell me that I have difficulties, but they don’t understand anything. They don’t listen to me because they don’t have time. At school I say I don’t understand, and they don’t include me, at home they don’t understand me, and my friends don’t understand me, they say I’m bad. I am fed up!/I am bored, I don’t like what they make me do/Everything is fine outside the school, with my classmates outside I am fine.
- Facilitator: Is anybody missing here? What about John’s friends’ point of view? (The facilitator proposes to consider also other people who have been mentioned but not staged).
- Friends: John teases me, beats me and bites me. We are all desperate. When he is not at school, it is much better.
- Friend’s parents: My son isn’t able to carry out the school curriculum as I wish, because of this child. My son is left behind because of him/Sometimes he is violent, swears at his classmates and his behaviour is inappropriate (Fig. 1).

Before moving further, the group took stock and the audience was invited to share observations, feelings, and comments that were recorded as important elements of the reflective process. In the following lines, the main observations are presented:

- This is real stuff. The school is at social services asking for help in involving the family. While we organize our work, we do not see children, as if they disappear in the eyes of adults.
- We waste too much time in organizing everything, we talk a lot, and we listen to a lot of information that is often confused or unclear, wasting time just talking and analysing and doing nothing in concrete.
- Who is coordinating the process? Who puts all the pieces together?
- All the actors seem to be alone, by themselves and not really connected with others.
- We don’t include the child. He is not participating; he is separated and far from the world of adults.
- We could save much time and energy if we worked more with families and if we organized community meetings with all the families for all the families, and not just focusing on children and families in care.
- What about his father? It this household a single-parent (mum) family or is John’s father not included? How to promote his participation? We often say fathers are “invisible” … but we poorly plan actions to involve them.

In order to enable participants to start and integrate the contents of the sociodramatic exploration into the innovation plan of the LabT, and to conclude the sociodrama session, the facilitator uses an integration of the techniques of sculpture and future projection. The main actors are invited to silently create a sculpture that shows a little improvement of situation in the near future. This aims at allowing actors to move in silence and to find a comfortable position according to their feelings, to the closeness and physical connection they like to have with others. They moved for a few minutes time and some actors change their position in relation to the movements of others. When the sculpture is done, every actor is invited to express how she/he feels in the new situation.

**Fig. 2:**
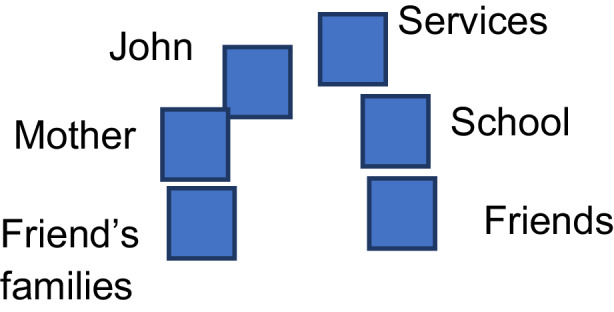
Position of the actors (second part)

- Service—Social worker: My idea is to work in involving all families and not just the family in the case or when they have a problem
- Friend’s families: I want to meet John and his family, but I need to understand more about this situation.
- John—the child: Let’s see what they want to do! What does the teacher want from me? I’m waiting, let’s see.
- School: The school is responsible for John and the community around him.
- Friends: We will wait for what the teachers tell us. Can anyone help us?
- Family—Mother: I’m here next to my child, John. His classmate’s families tell me they want to understand but they are only making trouble in this way. There is nothing to understand, I don’t want to be judged. I would like them to move closer to me and to look at me when they are close to me, and not to point me out and gossiping about my situation. (Fig. 2).

The sociodrama activity ended by de-roling, sharing and summing up of the main contents. The facilitator and the participants identified that the enactment showed the family-school-service cooperation was still work in progress and that different actors needed help in connecting one to the other and in community building. Throughout the session the group made space on the stage just for one family and its own situation. Queries arose about the families in the community and other children. Emphasis was put on the fact that full cooperation between the school and social services is a key factor in empowering the participation of all the families and not only families in care.

The LabT members were able to examine, in depth, their intention of working on community building and confirmed their willingness to organize and design their innovation plan on that topic.

## Discussion

By using sociodrama as a research technique, the session allowed more people to take part in comparison with other techniques, such as focus groups.

In a spontaneous way, the enactment process facilitated the exploration of those topics referred to and the questions identified by the LabT from the concerns held in the dossier: family participation, the role of case manager, of the school and of teachers in the multidisciplinary team.

The group explored to what extent their work is fragmented and siloed, how it is organized by a service-focused attitude rather than a child-focused, and by a single family focus rather a community one, with the consequences of not fostering community building and community capacity (Chaskin 2008; Ius 2020a).

The first part of the sociodrama allowed participants to safely interact and to represent relationships that resulted in “conserve”. Participants gave voice and action in a spontaneous way to the dynamics representing “how they are, how they work with families and mostly what are the dynamics between them”.

The first two voices were characterized by blaming the work other professionals in the microsystem with the family (the school VS the services stressing what they should do) and talking generically about the “child”. It is when somebody suggested to consider the commitment in a plural way (“we are facing …”; “we need to take …”) that the actors started to change attitude moving to a mesosystemic level of discussion: the verb “to bridge” addressed the focus on the family and the child was mentioned with his name, John. The following voices showed a more comprehensive attitude towards family and focused on the how to foster mother’s participation and her understanding of the situation of her child. It is interesting to observe that John was invited on stage after 14 expressions of the adults and by a suggestion of the facilitator: on the one hand, P.I.P.P.I. framework is structured on the “Child’s World” (Serbati et al. 2016) promoting a “children’s world centred” approach, on the other the dynamic on the stage shows professionals are likely to activate firstly an adult knowledge based dynamic, and only in a second moment they include children.

The involvement of John opened the sociodrama to a more communitarian approach. It fostered, indeed, the participation of other families and his peers. However, the dynamic got stuck in blaming John for his behaviour. Sharing their reflections, the participants confirmed the enactment mirrored the work process with families and with colleagues—including the difficulty to better involve fathers—and they underlined the necessity to have somebody, i.e. the case manager, to take care of the connection and the communication among all the people: family, teacher, service professionals, and community.

The sociodrama, allowed to focus on how social services’ cultures usually tend to deify conserves making professionals see practices and relationships among the parts in a rigid and self-confirming way that claims, “we do that, because we’ve always done it this way” or “you have the problem, you need to change this way, my job is helping you to change yourself”. We know that the same way of thinking can lead to the same old results, and above all it is unsuitable for complex situations because it does not foster transformation and innovation (Karp and Farral 2014).

Thanks to the reflections on the action enacted in the first part and through spontaneity, the second part gave space for a creative process that developed into a vision for the future to achieve for the LabT by means of the implementation plan. It is interesting to highlight that the enactment did not lead to an idyllic situation, which would have likely led to a conserve because of its stereotypical trait. It was dynamic and mostly it was generated by the participation of the child and mother and secondly of all the other actors who took part fully in the process according to their personality, knowledge, and expertise. From the point of view of building community capacity, the group of actors performed a situation in progression showing the need to participate and the responsibility of all them. While expressing the will to be more inclusive and nurturing community, they also expressed the need to know what to do, how to do it, and also to be helped to it. Moreover, the enactment drew the professionals’ attention to their essential role in activating and promoting participation and responsibility to people they work with. This is key to promote the meeting of children’s needs, to support people in need, and mostly to build a community of equity and support for all the members. The group used its creativity to gain self-understanding (Wilson 2017) and to co-created knowledge through a participative process where all participants gave a contribution in assessing what was happening and in planning the next expected outcomes, also giving prominence to interpersonal attention and open concerns. This knowledge refers to phronesis by Aristotle and, being a knowledge guiding decision making and following action, it seemed particularly appropriate to LabT’s goal. This goals are understanding the situation of the district and to decide the orientation for social innovation in it, in order to reach a meaningful change and development by making theory, research, and practice interact, and by a collective and collaborative endeavour (Freire 1970).

Feedback and sharing by professionals revealed their satisfaction about experiencing a research context that was deeply connected and coherent with the training and the participation in the programme P.I.P.P.I. Being part of a context that encourages the embodiment of actions, thoughts, and emotions, and not only of verbal, cognitive-reflective processes, enables participation. Moreover, by experiencing different positions and giving word and meaning to their movement, participants trained their empathy and their roles, according to Morenian perspective, having the chance “to develop an awareness of the subtle orchestration of thinking, feeling, action and context in professional encounters” (Walton 2012, p. 727).

Finally, by having an active role and by their enactments, they acknowledged themselves and the dynamics of the services they work in, so that they were able to consider the experience of research as an occasion and a step of Kolb’s cycle of experiential learning (Kolb 1984).

## Conclusion

The research experience presented in this paper shows that the use of sociodrama and associated techniques is consistent with participatory process connecting research, training and intervention in groups of social professionals working with vulnerable families.

From the theoretical point of view, Moreno’s Canon of Creativity is an interesting contribution to integrate “art” and “creativity” into a research perspective (ABR).

If, on the one hand, art based techniques and, even more so, sociodrama techniques are noteworthy modalities for involving participants in a hilarious and thought provoking manner, on the other hand they can integrate the rigorous method a research approach requires, with the spontaneity of participants that is key for breaking stereotypes and rigidity in response to research questions.

Moreover, there is no doubt that this method is consistent with the Participative and Transformative Evaluation method, because it provides researchers and participants a method and a set of techniques that at the same time meets the expectations of research and promote transformative practice. Social professionals have high rate in risk of compassion fatigue and burnout because of the difficult situations of families they work with (Fingley 1995; Teater and Ludgate 2014). Therefore, a “creative art approach” to social work is critical not only for providing them with new tools to foster people’s wellbeing and resilience, but also as a self-care opportunity to promote their own compassion satisfaction and resilience (Ius and Moretto 2017; Ius 2020b; Ius and Sidenberg 2017).

The role of the researcher, such as the facilitator, who guides the group research exploration and trains spontaneity to activate creativity leading to a new conserve is of paramount importance. His role is also meant as a function of scaffolding creative expression, according to the socio-cultural perspective (Vygotsky 1980).

On the other hand, imposing the group a predefined set of questions might risk fostering capture in the “research institution” rather than a participatory experience of “liberation” of collective and community.

For this reason, combining more common and conventional forms of academic knowledge and research with embodied, subjective, participatory practice knowledge, and research could nurture the integration between research and practices both on the academic side, and the field side. A philosophical reference for a future step regarding the theoretical framework may be explored within the existential phenomenology by making the Merleau-Ponty’s work on the concept of embodiment (Merleau-Ponty 1945) relate to the Morenian perspective on body, enaction, and encounter.

As it is argued about “the extent to which arts thinking has the potential to contribute not only to interpersonal work but also in radical paradigm development for social work” (Walton 2012, p. 727). In the future, it would be interesting to keep exploring how, besides group work practice, sociodrama and psychodrama can contribute to research paradigm in social work.

As a limitation of this experience the absence of families in the session must be underlined. The involvement of families within the sociodramatic process in research could be promoted. In fact, the method presented and discussed in this paper would be appropriate to encourage family participation, by harmonizing family-social services-school-researchers power dynamic and by offering a “potential stage” for creating participative research.

## Footnotes

1 Since the 7th edition has just finished and it is under analysis, and the 8th edition will conclude in spring 2021, data refer to 31 children and 29 families who took part in the 3rd and 6th edition of P.I.P.P.I.
